# The response of muscle protein synthesis following whole‐body resistance exercise is greater following 40 g than 20 g of ingested whey protein

**DOI:** 10.14814/phy2.12893

**Published:** 2016-08-10

**Authors:** Lindsay S. Macnaughton, Sophie L. Wardle, Oliver C. Witard, Chris McGlory, D. Lee Hamilton, Stewart Jeromson, Clare E. Lawrence, Gareth A. Wallis, Kevin D. Tipton

**Affiliations:** ^1^ Health and Exercise Sciences Research GroupUniversity of StirlingStirlingScotland; ^2^ Exercise Metabolism Research GroupDepartment of KinesiologyMcMaster UniversityHamiltonOntarioCanada; ^3^ GlaxoSmithKline Consumer HealthcareBrentfordUK; ^4^ School of Sport, Exercise and Rehabilitation SciencesUniversity of BirminghamBirminghamUK

**Keywords:** Fractional synthesis rate, intracellular signaling proteins, lean body mass, protein dose‐response

## Abstract

The currently accepted amount of protein required to achieve maximal stimulation of myofibrillar protein synthesis (MPS) following resistance exercise is 20–25 g. However, the influence of lean body mass (LBM) on the response of MPS to protein ingestion is unclear. Our aim was to assess the influence of LBM, both total and the amount activated during exercise, on the maximal response of MPS to ingestion of 20 or 40 g of whey protein following a bout of whole‐body resistance exercise. Resistance‐trained males were assigned to a group with lower LBM (≤65 kg; LLBM *n* = 15) or higher LBM (≥70 kg; HLBM *n* = 15) and participated in two trials in random order. MPS was measured with the infusion of ^13^C_6‐_phenylalanine tracer and collection of muscle biopsies following ingestion of either 20 or 40 g protein during recovery from a single bout of whole‐body resistance exercise. A similar response of MPS during exercise recovery was observed between LBM groups following protein ingestion (20 g – LLBM: 0.048 ± 0.018%·h^−1^; HLBM: 0.051 ± 0.014%·h^−1^; 40 g – LLBM: 0.059 ± 0.021%·h^−1^; HLBM: 0.059 ± 0.012%·h^−1^). Overall (groups combined), MPS was stimulated to a greater extent following ingestion of 40 g (0.059 ± 0.020%·h^−1^) compared with 20 g (0.049 ± 0.020%·h^−1^; *P* = 0.005) of protein. Our data indicate that ingestion of 40 g whey protein following whole‐body resistance exercise stimulates a greater MPS response than 20 g in young resistance‐trained men. However, with the current doses, the total amount of LBM does not seem to influence the response.

## Introduction

The stimulatory effects of resistance exercise and amino acid provision on muscle protein synthesis (MPS) are well‐documented (Biolo et al. 1997; Tipton and Wolfe 2004). Whereas amino acid provision from protein feeding alone stimulates MPS above basal rates (Morton et al. 2015; Witard et al. 2016), the combination of amino acid provision and resistance exercise results in greater stimulation of MPS than amino acid provision alone (Biolo et al. 1997). The type of protein ingested, timing of protein ingestion and amount of protein ingested in any given serving influence the response of MPS following exercise (Witard et al. 2016). Of these factors, the amount of protein ingested following exercise seems to have the most impact on MPS during recovery from resistance exercise. Hence, there is continuing interest in determining the factors that may influence the dose of ingested protein required to stimulate maximal MPS during exercise recovery while limiting significant amino acid oxidation.

Based on available evidence, a 20–25 g dose of high quality protein is considered sufficient to maximally stimulate MPS after resistance exercise in young adults (Morton et al. 2015; Witard et al. 2016). Results from the seminal study by Moore et al. (2009) demonstrated that ingestion of 40 g of egg protein following bilateral leg resistance exercise stimulated a similar response of MPS compared with 20 g of egg protein. We replicated these findings, detecting no statistically significant difference in the response of MPS to unilateral leg‐only resistance exercise after ingesting 20 or 40 g of whey protein in resistance‐trained young men (Witard et al. 2014). Taken together, these data support the recommendation that ingesting ~20–25 g of high‐quality protein after exercise will maximize stimulation of MPS during recovery from leg‐only resistance exercise.

One attribute that may influence the response of MPS to ingested protein following exercise is total muscle mass. It is often assumed that young adults possessing greater amounts of muscle mass require a larger dose of protein for maximal stimulation of MPS compared with young adults that possess less muscle mass (Churchward‐Venne et al. 2012b; Morton et al. 2015; Witard et al. 2016). Therefore, it may be necessary for individuals with greater muscle mass to ingest greater amounts of protein for maximal stimulation of MPS. Consequently, it seems intuitive to propose that the uptake of amino acids by a greater amount of muscle mass may be limited by a given amount of ingested protein. An extension to the notion that total muscle mass influences the dose response of MPS to protein ingestion is that the total amount of muscle involved in the exercise bout also will influence the MPS response. Prior resistance exercise increases blood flow to skeletal muscle, resulting in increased delivery and transport of amino acids into the activated muscle (Biolo et al. 1997). Thus, more exogenous amino acids would be necessary to ensure that delivery of amino acids to any given muscle would not be limiting. This concept is intuitively satisfying, but to our knowledge no study has directly investigated the influence of muscle mass on the response of MPS to protein ingestion. Therefore, in the present study we examined the response of MPS to two doses of whey protein ingested following exercise involving a greater amount of muscle mass, that is, a whole‐body exercise routine. We intended to maximize the difference in the amount of muscle exercised and the potential influence of muscle mass, both exercised muscle and muscle not involved in the exercise bout, on the response of MPS to protein ingestion.

The primary aim of the present study was to determine the response of MPS to two different doses of protein following a bout of whole‐body resistance exercise in resistance‐trained males with a large amount of LBM compared to those with a smaller amount of LBM. We hypothesized that the group with higher LBM would require more protein for maximal stimulation of MPS during recovery from whole‐body resistance exercise compared with the group with lower LBM.

## Methods

### Participants

Thirty healthy, resistance‐trained (≥2 sessions per week for previous 6 months) males participated in the present study and were grouped according to LBM. Fifty‐six participants were recruited and those that possessed LBM ≤ 65 kg were categorized as the lower lean body mass (LLBM) group (*n* = 15) and LBM ≥ 70 kg were categorized as the higher lean body mass (HLBM) group (*n* = 15). Volunteers with LBM between these values were not eligible to participate in the present study. The current study conformed to the standards of the latest version of the Declaration of Helsinki (2013) and the NHS West of Scotland Ethics Committee (REC number 12/WS/0316) approved the study. The nature of the study and its associated risks were explained to the participants in lay terms before informed written consent was obtained.

### Experimental design

In a two‐group, randomized, double‐blind, crossover design, each volunteer participated in two infusion trials designed to measure the response of MPS following whole‐body resistance exercise and whey protein ingestion. Trials were separated by ~2 weeks. Each infusion trial included the ingestion of either 20 or 40 g of whey protein isolate (GlaxoSmithKline, Brentford, UK) as a 500 mL drink immediately after exercise. The order of infusion trials, and thus dose of ingested protein, was randomized and an independent investigator prepared the drinks.

### Preliminary testing

Prior to study inclusion, participant LBM was assessed using dual‐energy x‐ray absorptiometry (DEXA) (GE Healthcare Systems, Hertfordshire). Participants with either ≤65 kg LBM or ≥70 kg LBM were included in the study. Each participant's one repetition maximum (1 RM) was assessed using a previously validated protocol (Baechle and Earle 2008) on select resistance exercise machines (Cybex International, MA); chest press, *latissimus* pull‐down, leg curl, leg press, and leg extension in this order. All leg exercises were carried out unilaterally, that is, on one leg at a time. Participants returned ~1 week later (at least 3 days prior to the first infusion trial) to confirm their 1 RM. Participant characteristics for each group are presented in Table 1.

**Table 1:** Participant characteristics

	LLBM (≤65 kg lean body mass)	HLBM (≥70 kg lean mass)
Age (y)	21.3 ± 2.2	23.2 ± 3.5
Body mass (kg)	76.8 ± 4.8	98.0 ± 7.8*
Height (m)	1.78 ± 0.05	1.84 ± 0.05*
Lean body mass (kg)	59.3 ± 3.9 (Range = 51.0‐64.4)	76.9 ± 4.3* (Range = 70.7‐83.9)
Fat mass (kg)	14.0 ± 3.3	17.0 ± 5.8
Lean mass (%)	77.7 ± 3.6	78.4 ± 4.7
Fat mass (%)	18.8 ± 3.7	17.3 ± 4.9
Appendicular lean mass (kg)	28.1 ± 2.1	37.4 ± 2.3*
1RM leg press (right leg) (kg)	126.0 ± 21.8	159.0 ± 29.5*
1RM leg press (left leg) (kg)	123.6 ± 23.9	158.7 ± 29.1*

Values are means ± SD. LLBM, lower lean body mass group; HLBM, higher lean body mass group.

*Significant difference from LLBM (*P* < 0.05).

### Dietary and activity control

Each participant completed a 3 days weighed food diary that was analysed using the nutritional analysis software package Wisp Version 4.0 (Tinuvel Software Systems, Anglesey, UK). Each participant's control diet was based on the self‐recorded intakes of the participants and matched the energy intake and composition of their habitual diet (Table 2). Diets were tailored to the individual's food preferences and were supplied in food packages for a 48 h period prior to both infusion trials. Participants completed a 7 days activity diary and were asked to keep their activity consistent during the study period. Participants were instructed to refrain from strenuous exercise 48 h before the infusion trials.

**Table 2:** Habitual macronutrient and energy intake of 30 trained, male weightlifters

	LLBM (≤65 kg lean mass)	HLBM (≥70 kg lean mass)
Energy intake (kcal·day^−1^)	2498 ± 676	2851 ± 619*
CHO intake (g·kg^−1^·day ^−1^)	3.5 ± 1.5	3.2 ± 1.2
CHO intake (% EI)	42 ± 14	37 ± 11
Protein intake (g·kg^−1^·day^−1^)	2.0 ± 0.5	1.9 ± 0.6
Protein intake (% EI)	23 ± 9	25 ± 6
Fat intake (g·kg^−1^·day^−1^)	1.0 ± 0.3	0.9 ± 0.2
Fat intake (% EI)	31 ± 12	30 ± 8

Values are means ± SD. Habitual diet calculated from 3 day diet records. LLBM, lower lean body mass; HLBM, higher lean body mass; CHO, carbohydrate; EI, energy intake.

*Significantly different from LLBM (*P* < 0.05).

### Experimental protocol

**Figure 1:**
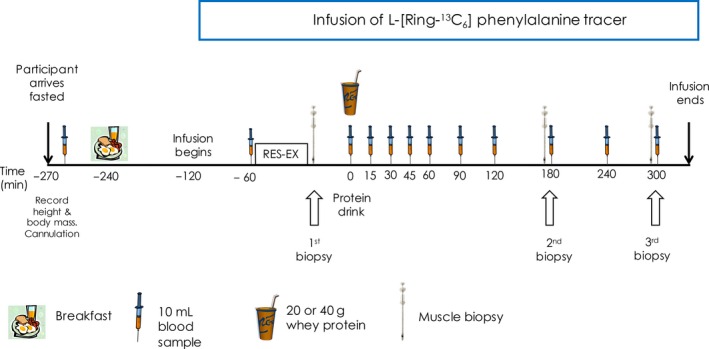
Schematic diagram of infusion trial protocol.

A schematic diagram of the experimental protocol is presented in Figure 1. Participants arrived at the research laboratories of the Health and Exercise Sciences Research Group at the University of Stirling at ~0600 h after an overnight fast. Upon arrival body mass was measured before a 20‐gauge cannula was inserted into a forearm vein and a fasted blood sample was collected. Participants were then provided with a standardized breakfast (7 kcal·kg^−1^ body mass) consisting of 50% of energy as carbohydrate, 30% of energy as protein and 20% of energy as fat. After breakfast participants rested in a semisupine position for 2 h before a primed constant infusion (0.05 μmol·kg^−1^·min^−1^; 2.0 μmol·kg^−1^ prime) of *L‐* [*ring*‐^13^C_6_] phenylalanine (Cambridge Isotope Laboratories, MA) was initiated through a 0.2 μm filter. Another 20‐gauge cannula was inserted into a vein on the contralateral hand or distal portion of the arm for frequent blood sampling. The cannula was periodically flushed with 0.9% saline solution and the arm was wrapped in a heated blanket to allow arterialized blood sampling. Approximately 1 h after starting the infusion participants performed an acute bout of resistance exercise on the following machines in the following order; chest press, *latissimus* pull‐down, leg curl, leg press, and leg extension. Leg exercises were performed for both legs unilaterally, that is, one leg at a time. Participants worked at 75% of their 1 RM at a cadence of 1 sec concentric – 2 sec eccentric contraction. Each participant was instructed to complete three sets of 10 repetitions with a final fourth set to volitional failure, to ensure that each participant was working at the same relative intensity. The exercise bout for the second trial was matched to the first. There were no significant differences between trials in volume (workload × repetitions) performed for any of the exercises.

Immediately after exercise, a skeletal muscle biopsy was obtained from the *vastus lateralis* under sterile conditions and local anaesthesia (2% lidocaine) using a 5 mm Bergstrom needle modified for manual suction. Participants then consumed a drink that contained either 20 or 40 g of a whey protein isolate made up in 500 mL of water (*t = *0 min). Drinks were enriched to 6% with *L‐* [*ring*‐^13^C_6_] phenylalanine tracer. Subsequent muscle biopsies were obtained from the same leg at 180 and 300 min. During the second trial the participants consumed the alternate dose of protein from the first trial and biopsies were obtained from the contralateral leg. Arterialized blood samples were obtained at *t = *−60, 0, 15, 30, 45, 60, 90, 120, 180, 240 and 300 min. The infusion was stopped following collection of the final blood sample at 300 min. Muscle samples were cleaned with ice cold 0.9% saline solution and were blotted, removing any blood, fat or connective tissue before being frozen in liquid nitrogen and stored at −80°C for further analysis. Blood samples were dispensed into EDTA‐ and sodium heparin‐ containing vacutainers and centrifuged at 2500 *g* for 15 min at 4°C. Plasma was extracted into ~0.5 mL aliquots and stored at −80°C for further analysis.

### Plasma analysis

Plasma samples were analysed for leucine, phenylalanine, and threonine concentrations using the internal standard method, as well as phenylalanine and tyrosine enrichments using gas‐chromatography, mass‐spectrometry (GCMS) as previously described (Witard et al. 2014). Briefly, plasma samples were thawed before adding acetic acid (1:1 dilution) and internal standard (U‐[^13^C_6_] leucine 0.52 mmol·L^−1^; U‐[^13^C_9_^15^N] phenylalanine 0.50 mmol·L^−1^; U‐[^13^C_4_^15^N] threonine 0.58 mmol·L^−1^). Next, amino acids were extracted and purified on cation‐exchange columns (Dowex 50WX8 hydrogen form 100–200 mesh resin, Sigma Aldrich, Dorset). Samples were dried under N_2_ gas before being converted to their tert‐butyl dimethylsilyl derivative (MTBSTFA). Finally, 2 μL of derivatized sample was injected into the GCMS (Agilent, Santa Clara, CA). Ions were monitored at m/z 302/308 for leucine, 336/346 and 234/240 for phenylalanine, 404/409 for threonine and 466/472 for tyrosine in split mode (1:50 split ratio).

Plasma urea concentrations were measured at each time point using an automated laboratory analyser (Instrumentation Laboratory, Milano).

### Muscle analysis

Muscle samples (30–35 mg) were homogenized in 500 μL 0.6 mol L^−1^ perchloric acid (PCA) prior to centrifugation at 3500 *g*. The supernatant was collected and a further 500 μL 0.6 mol L^−1^ PCA was added and was spun at 4500 rpm. This step was repeated. The resulting accumulation of supernatant had internal standard (U‐[^13^C_6_] leucine 0.01 mmol·L^−1^; U‐[^13^C_9_^15^N] phenylalanine 0.01 mmol·L^−1^) added to it. The supernatant and internal standard were added to the cation‐exchange columns and analysis continued as described above for plasma. Finally, 4 μL of intracellular (IC) sample was injected into the GCMS (same conditions as plasma analysis detailed above) and was run in splitless mode. IC leucine was detected at m/z 302/308 and phenylalanine (concentration and enrichment) at m/z 336/342 and 336/346.

Following IC extraction from the muscle sample the protein pellet was rinsed with doubly distilled H_2_O before being further homogenized in homogenization buffer (7.5 μL·mg^−1^ muscle; 50 mmol L^−1^ Tris‐HCl, 1 mmol L^−1^ EDTA, 1 mmol L^−1^ EGTA 10 mmol L^−1^ *β*‐glycerophosphate, 50 mmol L^−1^ NaF). Samples were spun and rinsed, removing the supernatant between spins. Thereafter, 0.3 mol L^−1^ NaOH was added and samples were heated at 50°C for 30 min with periodic spinning. The supernatant was collected and 0.3 mol L^−1^ NaOH was added to the pellet and spun before the supernatant was added to the previous collection. Next, 1 mol L^−1^ PCA was added to supernatant and spun. The resulting supernatant was removed and discarded; the remaining pellet was rinsed twice in ethanol. The pellet was hydrolysed overnight at 110°C in 0.5 mol L^−1^ HCl and 1 mL of activated resin. The hydrolysed samples were purified on the cation‐exchange columns and dried under N_2_. Samples were converted to their n‐acetyl, n‐propyl ester (NAP) derivative. Finally, 1 μL of derivatized sample was injected into a gas‐chromatography combustion isotope ratio mass spectrometer (Thermo Finnigan, Hertfordshire, UK) and run in splitless mode monitoring m/z 44/45 carbon ratio.

### p70S6K1 activity assays

Muscle tissue (~30–50 mg) was homogenized in a 10‐fold volume of homogenization buffer (50 mmol L^−1^ TrisHCl pH 7.5, 0.1 mmol L^−1^ EGTA, 1 mmol L^−1^ EDTA, 1% (v/v) TritonX‐100, 50 mmol L^−1^ NaF, 5 mmol L^−1^ NaPPi, 0.27 mol L^−1^ sucrose, 0.1% *β*‐mercaptoethanol, 1 mmol L^−1^ Na_3_(OV)_4_, and 1 Complete (Roche) protease inhibitor tablet per 10 mL) using dounce homogenization. Samples were clarified by centrifugation at 4°C for 45 min at 21,000 × *g*. The protein concentration of each sample was quantified using the DC protein assay (BioRad, Hertfordshire, UK) and Gen 5 software (BioTek, Vermont) according to the manufacturers’ instructions. Total p70 ribosomal S6 kinase 1 (p70S6K1) was immunoprecipitated from the lysate for 2 h at 4°C in homogenization buffer with 4 μg p70S6K1 antibody (Santa Cruz Biotechnology Inc, Heidelberg, Germany). Activity assays were then performed as previously described (McGlory et al. 2014).

### Calculations

Myofibrillar fractional synthesis rate (FSR) was calculated using the standard precursor product equation below:

$$\text{FSR}=[\left(E_{B2}-E_{B1}\right)/\left(E_{\mathrm{IC}}\times t\right)]\times 100$$

where *E*_B_ (B2 is the biopsy at the later time point, B1 is the biopsy from the earlier time point) is the enrichment of bound phenylalanine, *E*_IC_ is the IC phenylalanine enrichment of the biopsies and *t* is time of tracer incorporation (h). IC phenylalanine enrichment was used as the precursor in all FSR calculations.

Plasma and IC amino acid concentrations were calculated by the internal standard method:

$$C=Q_{\mathrm{is}}/V\times E_{\mathrm{is}}$$

where *Q*_is_ is the amount of internal standard added to the sample, *V* is the volume of plasma or IC water (663 mL·kg^−1^ muscle) (Biolo et al. 1995) and *E*_is_ is the internal standard tracer to tracee ratio in the plasma.

Whole‐body phenylalanine oxidation rates were estimated using the phenylalanine balance model based on the hydroxylation of *L‐* [*ring*‐^13^C_6_] phenylalanine to *L‐* [*ring*‐^13^C_6_] tyrosine, without measuring ^13^CO_2_ enrichment in the breath (Thompson et al. 1989):

$$P_{t}/P_{p}\times \left(Q_{p}^{2}/\left(E_{p}/E_{t}\right)-1\right)\times \left(F+Q_{p}\right)$$

where *P*_t_/*P*_p_ is the molar ratio of fluxes of tyrosine and phenylalanine, *Q*_p_ equals the rate of disappearance of phenylalanine under steady state conditions, *E*_p_ is the enrichment of phenylalanine, *E*_t_ is the enrichment of tyrosine and *F* equals the infusion rate.

Incremental area under the curve (AUC) for plasma amino acid and urea concentrations, as well as rate of phenylalanine oxidation, was calculated using GraphPad Prism (V6, Graphpad Software Incorporation, California). AUC of urea concentrations and phenylalanine oxidation rates was calculated from a baseline = 0 for each and for amino acid concentrations the baseline was calculated from the concentration at *t* = 0 min time point.

### Statistical analysis

Data were plotted in graphical format to assess normal distribution using Minitab Version 17.0 (Minitab Software Systems, State College, PA). Box cox transformations were performed on data that were not normally distributed. The statistical significance level was set at *P* = 0.05. Anthropometric, strength and dietary data (HLBM vs. LLBM) were analysed using one factor (group) ANOVA using SPSS (Version 21, IBM UK Ltd, Hampshire). Plasma amino acid and urea concentrations and phenylalanine oxidation rates were analysed using repeated measures ANOVA with dose (2 levels) and time (12 levels) as within‐factors and group (HLBM and LLBM) as a between‐factor. AUC for plasma amino acid and urea concentrations and phenylalanine oxidation rates were calculated and analysed using a repeated measures ANOVA with dose as a within‐factor and group as a between‐factor. Myofibrillar FSR and IC amino acid concentrations (leucine and phenylalanine) were analysed using a mixed model, repeated‐measures ANOVA in SPSS, with dose (2 levels) and time (3 levels) as within‐factors and group as a between‐factor. AUC was calculated for IC leucine and phenylalanine concentrations and analysed using a repeated measures ANOVA with dose as a within‐factor and group as a between‐factor. If any interaction was detected, Tukey's post‐hoc tests were performed using Minitab statistical software. Cohen's effect size (*d*) and 95% confidence intervals (CI) were calculated for group and dose. Effect sizes of 0.2 are considered small, 0.5 considered medium and >0.8 are considered large (Cohen 1988). If 0 was not contained within the confidence intervals for the effect size the effect was deemed significant.

## Results

### Blood and muscle intracellular amino acid concentrations

**Figure 2:**
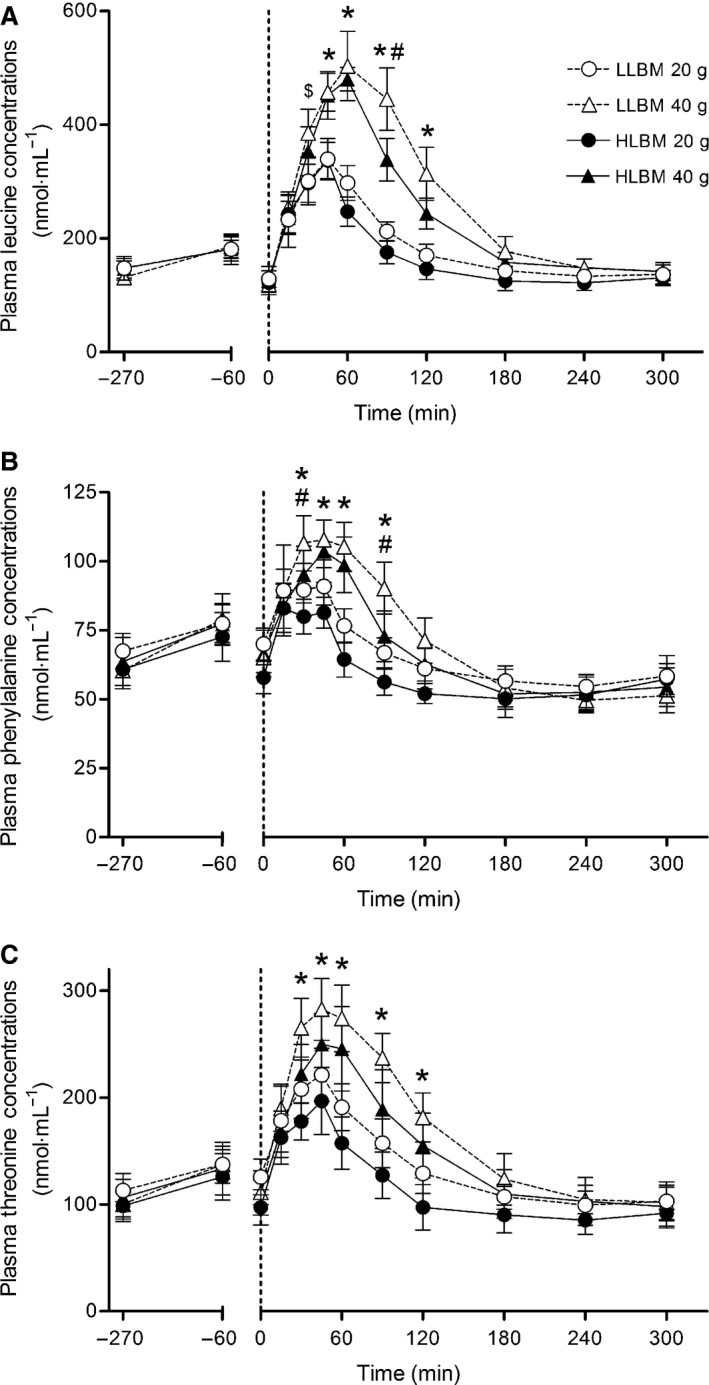
Plasma leucine (A), phenylalanine (B) and threonine (C) concentrations following ingestion of either 20 or 40 g of whey protein isolate in both the lower lean body mass (LLBM) and higher lean body mass (HLBM) groups. Data presented as means with 95% confidence intervals. *Significant difference between doses; ^#^significant difference between groups; ^$^significant difference between doses in LLBM group only (all *P* < 0.05).

Plasma leucine concentrations peaked at 45 min with 20 g of ingested whey protein and at 60 min with 40 g in both groups (Fig. 2A). Plasma leucine concentrations were higher at 45 (*d* = 1.81; CI = 1.21–2.42), 60 (*d* = 3.13; CI = 2.38–3.89), 90 (*d* = 2.64; CI = 1.94–3.33) and 120 min (*d* = 2; CI = 1.38–2.63) in both groups with 40 g compared with 20 g of whey protein whilst being elevated also at 30 min (*d* = 1.13; CI = 0.36–1.90) in LLBM. Plasma leucine concentrations for 40 g were higher in LLBM than HLBM at 90 min (*d* = 1.26; CI = 0.48–2.04) (group × time × dose interaction; *P* = 0.048). Plasma leucine concentrations, expressed as AUC after protein ingestion, were greater with 40 g (70,579 ± 7620 nmol·mL^−1^ × 300 min) compared with 20 g of whey protein (49,108 ± 6516 nmol·mL^−1^ × 300 min) (*d* = 2.68; CI = 1.98–3.38). With 40 g plasma leucine AUC was 1.3‐fold greater in the LLBM (74,630 ± 6122 nmol·mL^−1^ × 300 min) than HLBM (66,526 ± 6901 nmol·mL^−1^ × 300 min) (*d* = 1.42; CI = 0.62–2.22) (dose × group interaction; *P* = 0.039) (data not shown).

Plasma phenylalanine concentrations were greater with 40 g compared with 20 g of ingested whey protein at 30 (*d* = 0.94, CI = 0.40–1.47), 45 (*d* = 1.33, CI = 0.77–1.89), 60 (*d* = 2.08; CI = 1.46–2.71), and 90 min (*d* = 1.32; CI = 0.76–1.36) in both groups (dose × time interaction; *P* < 0.001) (Fig. 2B). Although not statistically significant the effect size between doses of ingested whey protein at 120 min (*d* = 0.83; CI = 0.29–1.36) was large. At 30 (*d* = 0.58; CI = 0.06–1.09) and 90 min (*d* = 0.83; CI = 0.30–1.36), regardless of protein dose, plasma phenylalanine concentrations were greater in LLBM than HLBM (time × group interaction; *P* = 0.021). Plasma phenylalanine concentrations, expressed as AUC after protein ingestion, were greater in LLBM with 40 g (1552 ± 2187 nmol·mL^−1^ × 300 min) compared with 20 g of ingested whey protein (−1512 ± 2523 nmol·mL^−1^ × 300 min) (dose × group interaction; *P* = 0.022; *d* = 1.30; CI = 0.51–2.09) (data not shown).

Plasma threonine concentrations peaked at 45 min and were elevated in both groups at 30 (*d* = 1.04; CI = 0.50–1.58), 45 (*d* = 1.09; CI = 0.54–1.63), 60 (*d* = 1.54; CI = 0.96–1.93), 90 (*d* = 1.37; CI = 0.81–1.93), and 120 min (*d* = 1.22; CI = 0.66–1.77) in 40 g compared with 20 g of ingested whey protein (time × dose interaction *P* < 0.010) (Fig. 2C). Plasma threonine concentrations were higher in LLBM compared with HLBM (main effect of group; *P* = 0.022) but no interaction was observed. Plasma threonine concentrations, expressed as AUC after protein ingestion, were greater with 40 g (50,230 ± 11,713 nmol·mL^−1^ × 300 min) compared with 20 g of ingested whey protein (39,504 ± 8889 nmol·mL^−1^ × 300 min) (*d* = 1.68; CI = 1.09–2.27). Following ingestion of 40 g of whey protein plasma threonine AUC was greater in LLBM (53,302 ± 8682 nmol·mL^−1^ × 300 min) than HLBM (47,157 ± 13,732 nmol·mL^−1^ × 300 min) (*d* = 0.96; CI = 0.20–1.71) (dose × group interaction; *P* = 0.005) (data not shown).

Muscle IC leucine concentrations were greater with 40 g compared with 20 g at 180 (*d* = 0.57; CI = 0.05–1.09) and 300 min (*d* = 0.65; CI = 0.13–1.17) (dose × time interaction; *P* = 0.005) (Table 3). IC leucine concentrations, expressed as AUC, were greater with 40 g than 20 g (main effect of dose; *P* = 0.001; *d* = 0.82; CI = 0.30–1.35) and IC leucine concentrations in LLBM was greater than HLBM with both doses combined (main effect of group; *P* = 0.012; *d* = 0.57; CI = 0.05–1.08). There were no differences between groups or doses in IC phenylalanine concentrations (Table 3). IC phenylalanine concentrations were lower at 180 and 300 min compared with 0 min (main effect of time; *P* < 0.001) and AUC of IC phenylalanine concentrations was negative for all groups.

**Table 3:** Intracellular leucine and phenylalanine concentrations in response to ingesting 20 or 40 g of whey protein in low (LLBM) and high (HLBM) lean body mass groups

	Leucine				Phenylalanine			
	0	180	300	AUC	0	180	300	AUC
LLBM 20 g	128 ± 24	148 ± 42	149 ± 34	4380 ± 7555#	57 ± 11	49 ± 14	52 ± 13	−1478 ± 2572
LLBM 40 g	132 ± 26	176 ± 42*	159 ± 39*	8436 ± 7701*, #	56 ± 12	48 ± 13	50 ± 11	−1538 ± 2779
HLBM 20 g	145 ± 47	136 ± 44	124 ± 31	−1934 ± 6039	54 ± 10	46 ± 13	48 ± 14	−2194 ± 1608
HLBM 40 g	135 ± 28	159 ± 48*	160 ± 35*	5027 ± 6109*	58 ± 11	45 ± 15	50 ± 11	−2670 ± 1785

LLBM, low lean body mass group; HLBM, high lean body mass group, 20 g, twenty grams of ingested whey protein; 40 g, forty grams of ingested whey protein; 0, time of whey protein ingestion; 180, 180 min after whey protein ingestion, 300, 300 min after whey protein ingestion; AUC, area under the curve with baseline set as the concentration at the 0 min time point. Data expressed as nmol·mL^−1^ intracellular water (concentration) and nmol·mL^−1^ × 300 min (AUC) and values presented are means ± SD.

*Significant difference compared with 20 g dose; ^#^significant difference between groups at corresponding protein dose.

### Tracer enrichments

**Figure 3:**
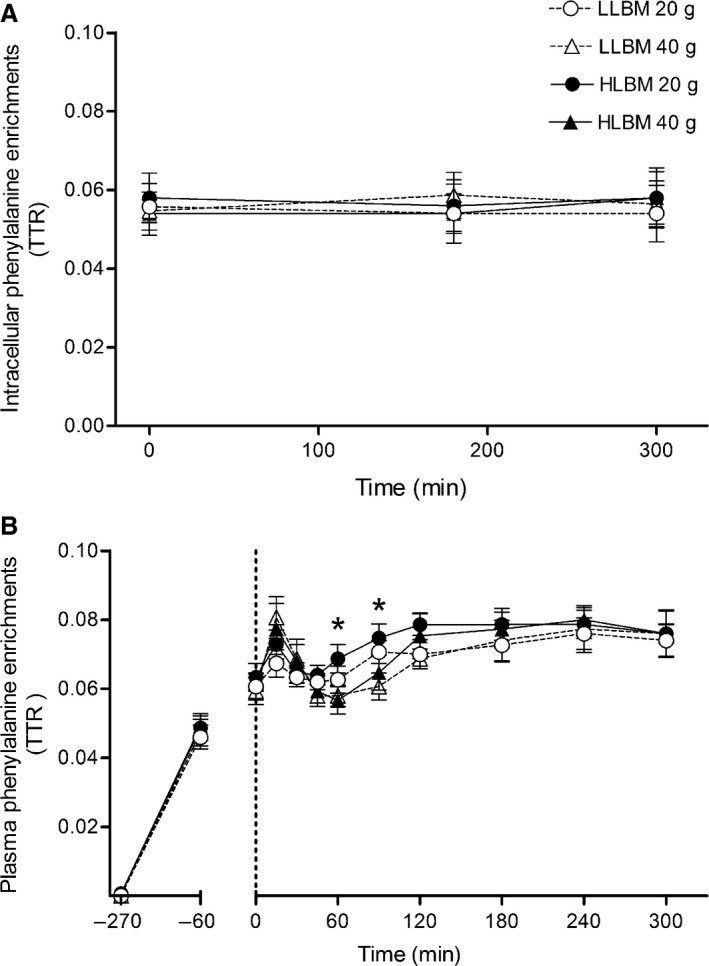
Muscle intracellular (A) and plasma (B) phenylalanine enrichments expressed over time during *L‐* [*ring*‐^13^C_6_] phenylalanine infusion in both the lower lean body mass (LLBM) and higher lean body mass (HLBM) groups. Data presented as means with 95% confidence intervals. Data expressed as tracer to tracee ratio (TTR). Ingestion of either 20 or 40 g whey protein isolate occurred at 0 min. *Significant difference between doses (*P* < 0.05).

No differences were observed in IC phenylalanine enrichments between groups or doses or across time (Fig. 3A). Plasma phenylalanine enrichments transiently increased immediately following protein ingestion (Fig. 3B). Nevertheless, when myofibrillar FSR was calculated using the AUC of plasma phenylalanine enrichments as precursor, the responses to the two protein doses in each group were not different from when FSR was calculated using IC phenylalanine enrichments as the precursor.

### Phenylalanine oxidation rates and plasma urea concentrations

**Figure 4:**
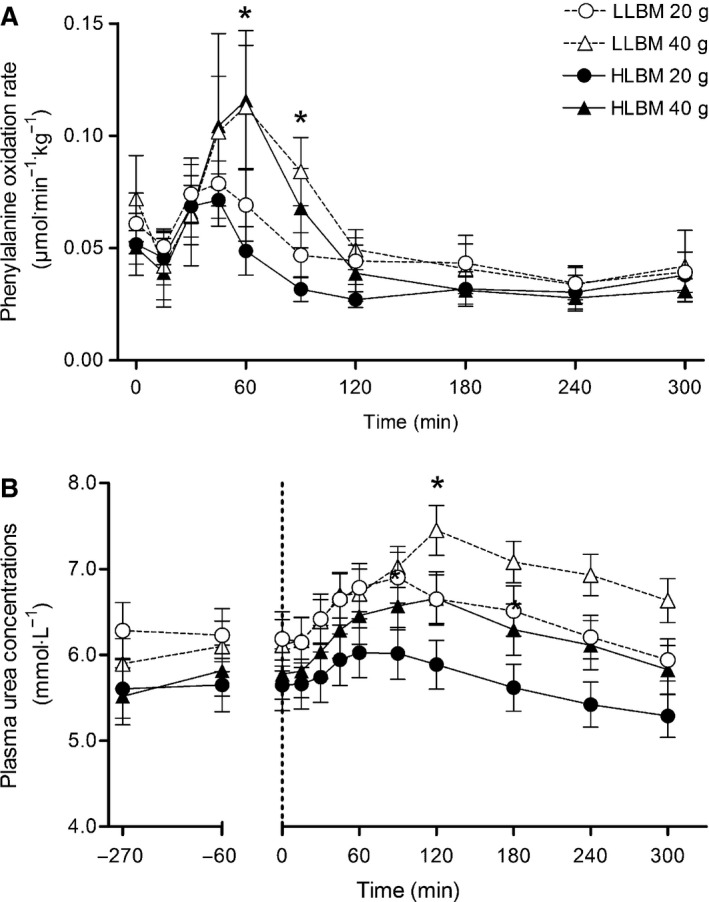
Rate of phenylalanine oxidation (A) and plasma urea concentrations (B) following ingestion of either 20 or 40 g of whey protein isolate in both the lower lean body mass (LLBM) and higher lean body mass (HLBM) groups. Data presented as means with 95% confidence intervals. *Significant difference between doses (*P* < 0.05).

The rate of phenylalanine oxidation was greater with ingestion of 40 g compared with 20 g of ingested whey protein at 60 (*d* = 1.35; CI = 0.78–1.91) and 90 min (*d* = 1.51; CI = 0.93–2.08) (dose × time interaction; *P* < 0.001) (Fig. 4A). There was a moderate effect between doses at 45 min (*d* = 0.62; CI = 0.11–1.14) but this effect was not statistically significant. Phenylalanine oxidation rates, expressed as AUC, were greater with ingestion of 40 g of whey protein (15.5 ± 5.6 μmol·kg^−1^·min^−1^ × 300 min) compared with 20 g (12.8 ± 3.8 μmol·kg^−1^·min^−1^ × 300 min) (main effect of dose; *P* < 0.001; *d* = 0.56; CI = 0.05–1.08) but there were no differences between groups (*P* = 0.068; *d* = 0.54; CI = 0.03–1.06).

Plasma urea concentrations were greater with ingestion of 40 g of whey protein compared with 20 g at 120 (*d* = 0.66; CI = 0.14–1.18), 180 (*d* = 0.55; CI = 0.03–1.06), 240 (*d* = 0.66; CI = 0.14–1.18), and 300 min (*d* = 0.58; CI = 0.07–1.10) (dose × time interaction; *P* < 0.001) (Fig. 4B). The AUC for plasma urea concentrations was greater in 40 g (1974 ± 317 mmol·L^−1^ × 300 min) compared with 20 g (1821 ± 320 mmol·L^−1^ × 300 min) (main effect of dose; *P* = 0.002, *d* = 0.48; CI = −0.03 to 0.99) and also in LLBM (2003 ± 292 mmol·L^−1^ × 300 min) compared with HLBM (1792 ± 327 mmol·L^−1^ × 300 min) (main effect of group; *P* = 0.047; *d* = 0.68; CI = 0.16–1.20).

### Myofibrillar muscle protein synthesis

**Figure 5:**
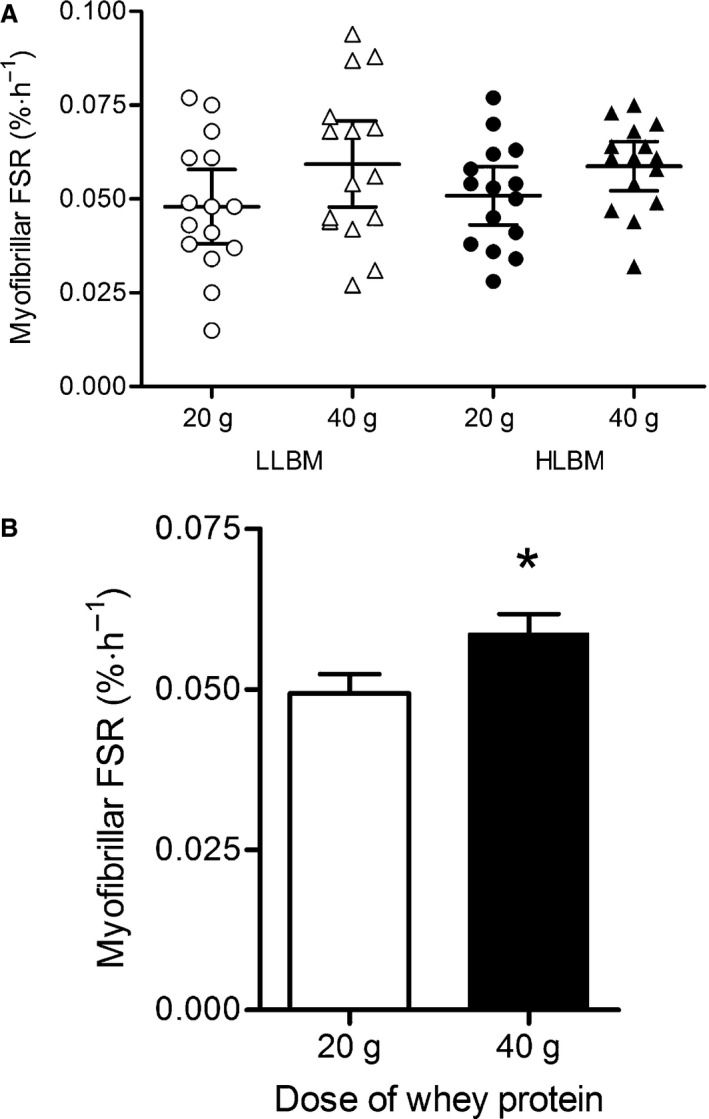
Myofibrillar fractional synthesis rate (FSR) presented for each individual participant following ingestion of either 20 or 40 g whey protein isolate in lower lean body mass (LLBM) and higher lean body mass (HLBM) groups (A). Line represents the mean for each condition. Mean ± SD of myofibrillar FSR following the ingestion of 20 and 40 g whey protein isolate for both groups combined (B). *Significant difference between doses with all participants of each group combined (*P* = 0.005). FSR was determined over the 0–5 h period following protein ingestion.

There was no significant interaction between protein dose and LBM group nor was there a statistically significant difference in myofibrillar FSR (determined for the entire 0–300 min incorporation period) between HLBM and LLBM groups (Fig. 5A). However, there was a main effect of protein dose for FSR with all participants of both groups combined (Fig. 5B). Overall, myofibrillar FSR was ~20% higher with ingestion of 40 g compared with 20 g of whey protein (*P* = 0.005; *d* = 0.59; CI = 0.08–1.11) following whole‐body resistance exercise, irrespective of group. There was no apparent time resolution of FSR following exercise. FSR determined for 0–180 min (*d* = 0.53; CI = 0.02–1.05) and 180–300 min (*d* = 0.50; CI = −0.02 to 1.01) following exercise followed a similar pattern to the overall time period (Table 4). As with the entire 300 min incorporation period, FSR was significantly greater for 40 g compared with 20 g at both the early (0–180 min) and later (180–300 min) time periods following protein ingestion.

**Table 4:** Myofibrillar fractional synthetic rates for 20 and 40 g trials at different times after protein ingestion

	20 g	40 g
0–180 min	0.0501 ± 0.0191	0.0613 ± 0.0243*
180–300 min	0.0471 ± 0.0218	0.0586 ± 0.0243*

Values are means ± SD. 0–180 min, first 180 min after ingestion of whey protein. 180–300 min, second period after ingestion of whey protein.

*Significantly different from 20 g at corresponding time period (*P* < 0.05).

### p70S6K1 activity

**Figure 6:**
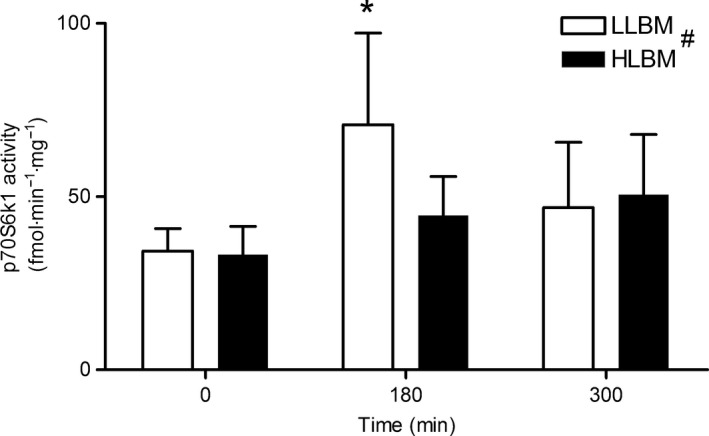
P70S6K1 activity following whey protein isolate ingestion (whey protein doses combined) in both the lower lean body mass (LLBM) and higher lean body mass (HLBM) groups. Data presented as means with 95% confidence intervals. *Significant difference from 0 min; ^#^main effect of group (*P* = 0.002) and main effect of time (*P* = 0.008).

There was no effect of protein dose on p70S6K1 activity, hence the data are presented with both doses combined (Fig. 6). The activity of p70S6K1 was higher in LLBM than HLBM regardless of time or dose (main effect of group; *P* = 0.002) although this difference appeared to be primarily driven by the 180 min time point. The effect sizes for each time point were as follows; 0 min *d* = 0.02; CI = −0.49 to 0.53; 180 min *d* = 0.48; CI = −0.03 to 0.99; 300 min *d* = −0.08; CI = −0.58 to 0.43. The activity of p70S6K1 was greater at 180 min compared with 0 min (main effect of time; *P* = 0.008), but there were no differences in p70S6K1 activity between 0 and 300 min time points or 180 and 300 min time points.

## Discussion

The primary aim of the present study was to investigate the response of MPS to two doses of protein following whole‐body resistance exercise in trained young males with higher and lower amounts of LBM. We hypothesized that individuals with greater LBM would require more protein for greater stimulation of MPS during recovery from whole‐body resistance exercise compared with individuals with lower LBM. Our novel findings demonstrate that, overall, ingesting a 40 g dose of whey protein isolate stimulated MPS to a greater extent than a 20 g dose of whey protein isolate during acute (0–300 min) exercise recovery in young, resistance‐trained males. However, contrary with our hypothesis, the response of MPS following whole‐body resistance exercise was similar in both groups of resistance‐trained males, despite different amounts of LBM.

The general consensus within the scientific literature is that ingestion of 20–25 g of protein after resistance exercise is sufficient for the maximal stimulation of MPS (Churchward‐Venne et al. 2012b; Morton et al. 2015; Witard et al. 2016). Previous studies in young, resistance‐trained males reported no statistically significant difference in the response of MPS to ingestion of 20 or 40 g of protein after resistance exercise (Moore et al. 2009; Witard et al. 2014). However, in the present study we demonstrated that ingestion of 40 g of protein significantly increased myofibrillar MPS compared to ingestion of 20 g of protein after resistance exercise. The reason our results and previous results (Moore et al. 2009; Witard et al. 2014) do not agree cannot be determined with absolute certainty, but methodological differences exist between studies that may offer some explanation. We believe the most likely explanation for the difference in response of MPS to resistance exercise and protein ingestion is the amount of muscle activated during the exercise bout. Whereas our participants performed a bout of whole‐body resistance exercise, those in Moore et al. (2009) and Witard et al. (2014) performed leg only exercise bouts (bilateral and unilateral, respectively). Accordingly, we suggest that the overall demand for amino acids following a bout of whole‐body resistance exercise is greater compared to a bout of unilateral or bilateral leg resistance exercise. Nutritive blood flow increases following resistance exercise (Biolo et al. 1995) facilitating the delivery of amino acids to the working muscle. Resistance exercise increases amino acid transport and uptake into the muscle (Biolo et al. 1995). Therefore, the greater the amount of muscle activated the greater the overall amount of amino acids taken up by muscle after exercise. Moreover, Pennings et al. (2011) demonstrated that incorporation of amino acids from exogenous protein for de novo MPS was greater in exercised than rested muscle. Consequently, MPS is higher in response to resistance exercise followed by protein feeding compared with feeding alone (Pennings et al. 2011; Witard et al. 2014). Thus, the greater the amount of muscle utilized during a resistance exercise bout, the greater the demand for amino acids that must be met by exogenous sources for MPS to be increased in any given muscle. Furthermore, blood flow is reduced to any given muscle when other muscles are activated compared to when one muscle group alone is exercised (Volianitis and Secher 2002), thus reducing amino acid delivery to any particular muscle. We propose that amino acid supply may have been insufficient with ingestion of 20 g whey protein to meet the demands of the exercised muscle during recovery from whole‐body resistance exercise. Thus, MPS in the measured muscle is lower when only 20 g of whey protein was ingested following whole‐body resistance exercise. Conversely, in the 40 g trial there were more amino acids available for all the exercised muscles and MPS measured in the legs likely was able to respond at a greater rate. Hence, whole‐body resistance exercise seems to lead to a broader dispersal of ingested amino acids and thus the stimulation of MPS by ingestion of 20 g of whey protein is limited by amino acid availability in some muscles compared to ingestion of 40 g of whey protein.

The notion that amino acid availability to any single muscle may be limited with whole‐body exercise may help explain the seemingly low FSR values that we report in the present study. The mean FSR values are approximately 71% and 76% of the FSR values for the 20 and 40 g, respectively, doses of whey protein that we reported previously (Witard et al. 2014). The lower FSR values in the present study seem to be consistent with the notion of reduced amino acid availability due to the whole‐body resistance exercise bout. Thus, whereas we cannot definitively state that the lower FSR values we observed in this study are due to the dispersal of available amino acids to a greater amount of muscle, it is an intuitively satisfying explanation. Moreover, this notion is consistent with the differential responses of MPS to the 20 and 40 g doses of protein we report following whole‐body, but not leg‐only, resistance exercise.

Although we believe the amount of muscle exercised is the most likely explanation for the differences in results observed between this study and previous studies (Moore et al. 2009; Witard et al. 2014), alternative nonphysiological explanations must be considered. One alternative explanation may be related to sample size. The present study results stem from *n* = 30 in a crossover design, whereas previously we (Witard et al. 2014), and others (Moore et al. 2009), recruited 12 and 6 participants (in each group), respectively. However, since both previous studies (Moore et al. 2009; Witard et al. 2014) observed a mean difference of ~10% in MPS between the 20 and 40 g conditions and we detected a difference of ~20% in the present study, there appears to be a real difference in the response of MPS to whole‐body resistance exercise plus protein ingestion and leg‐only exercise plus protein ingestion.

Other possible explanations for the different results between studies may relate to differences in the type of protein ingested following exercise and the fraction of muscle protein for which the muscle protein synthetic rate was determined. However, the results of Moore et al. (2009) and Witard et al. (2014) are similar despite these methodological differences. Furthermore, our present and previous (Witard et al. 2014) results differ despite similar methodologies. Thus, the factor that differs between the present study and previous studies (Moore et al. 2009; Witard et al. 2014) is the amount of muscle activated during the exercise bout. The postprandial time period for measurement of MPS is another potential factor that may explain the variable response to protein ingestion in these studies. Both previous studies assessed MPS over 4 h following protein ingestion (Moore et al. 2009; Witard et al. 2014). We measured MPS for a total of 5 h after protein ingestion. However, we also attempted to determine more time resolution of the MPS response by calculating FSR for two distinct time periods within the overall 5 h. MPS was greater for the 40 g dose than the 20 g dose for both time periods. Thus, it seems that the differences in time of measurement between studies is unlikely to explain the different responses to ingesting 40 and 20 g of whey protein that are reported. Therefore, whereas we cannot definitively determine the exact cause of the disparity in results between the present study and the previous research, it may be due to the whole‐body exercise performed.

The dose of protein necessary for maximal stimulation of MPS following resistance exercise often has been thought to be greater for those individuals with greater LBM (Churchward‐Venne et al. 2012b; Morton et al. 2015; Witard et al. 2016). However, our study is the first to directly address whether the total amount of LBM influences the MPS response to resistance exercise combined with protein feeding. Whereas we did not observe any influence of the amount of LBM on the MPS response, we did observe that 40 g of protein stimulated MPS to a greater extent than 20 g following whole‐body resistance exercise. Thus, it seems that the overall amount of muscle mass possessed by the individual is a less important determinant of the maximally effective dose of protein to ingest than the amount of muscle mass activated during exercise. However, it is possible that LBM may be an important determinant of the response of MPS to a given amount of protein in other circumstances. For example, if leg‐only exercise is performed, perhaps MPS would not be similar between individuals with higher and lower amounts of LBM in response to ingestion of 20 and 40 g of protein. Our data suggest that several factors other than the total amount of protein ingested may influence the metabolic response of muscle following exercise, that is, the physiological response of muscle to varying amounts of ingested protein likely is less simple than is often portrayed. More studies need to be performed to examine these complexities.

The mTORC1 signaling pathway regulates MPS (Kimball et al. 2002; Philp et al. 2011) and p70S6K1 activity is one of the key readouts of activation of the pathway (Hamilton et al. 2009). There were no differences in p70S6K1 activity between the 20 and 40 g trials in the present study, yet there was a significant difference in MPS between protein doses. These results are consistent with the findings of a previous study that observed no increase in p70S6K1 phosphorylation at 4 h in response to resistance exercise and increasing doses of egg protein despite increased MPS at higher doses (Moore et al. 2009). However, in the present study p70S6K1 activity was 1.6‐fold greater at 180 min following protein ingestion in the LLBM compared with the HLBM group. We believe that this difference in p70S6K1 activity was driven by the higher plasma leucine concentration in the LLBM compared with the HLBM group with ingestion of 40 g of whey protein. Leucine plays a unique role in the regulation of mTORC1 signaling and MPS in combination with exercise (Anthony et al. 1999; Kimball and Jefferson 2005, 2006; Apro and Blomstrand 2010; Moberg et al. 2014). However, this elevated activity was not associated with a greater response of MPS in LLBM compared to HLBM. Thus, in the context of both dose and LBM groups there appears to be a discrepancy between the response of p70S6K1 activity and MPS. This apparent disconnect between signaling and MPS has been noted in multiple studies previously (Dreyer et al. 2006; Mayhew et al. 2009; Witard et al. 2009; Atherton et al. 2010; Churchward‐Venne et al. 2012a). The discrepancy could be due to the temporal pattern of the response of mTORC1 signaling. It seems apparent that the coupling of MPS with molecular signaling is most certain only during the short temporal period around peak signals (Atherton et al. 2010). Moore et al. (2011) showed that whereas p70S6K1 phosphorylation was elevated above baseline at 180 and 300 min following resistance exercise and protein ingestion, the greatest response was at 60 min. Consequently, it seems likely that by measuring p70S6K1 activity at 180 and 300 min following exercise and protein ingestion we may not have measured the maximal response. Therefore, it is perhaps not surprising that differences in p70S6K1 activity do not directly correspond with differences in MPS in the present study.

In summary, our data show for the first time that ingestion of 40 g whey protein results in greater stimulation of MPS than 20 g whey protein following whole‐body resistance exercise in healthy, young males. Thus, our data show for the first time that 20 g of protein does not stimulate a maximal response of MPS in young, trained men. These data may suggest that whole‐body resistance exercise alters the dynamics of the MPS response to protein feeding compared with exercising a smaller amount of muscle, such as the case with lower‐limb exercise protocols (Moore et al. 2009; Witard et al. 2014). Whereas the total amount of muscle possessed by the individual does not seem to influence the MPS response in an individual muscle, the amount of muscle that has been exercised seems to be important at doses of 40 g whey protein and below. We conclude that more protein is necessary for the increased stimulation of MPS following whole‐body compared to unilateral or bilateral resistance exercise. However, we must stress that our data do not allow for a final explanation for the differences between our study and previous studies. Future studies should examine this issue directly. Thus, our results may have implications for the design and implementation of resistance exercise training programmes and feeding strategies to optimize muscle mass. At the very least, it seems clear that ingesting 20 g of protein does not maximally stimulate MPS following resistance exercise in all circumstances. Moreover, it is important to note that our results are limited to healthy, young resistance‐trained individuals. Other populations with differing metabolic responses to protein, for example, older individuals (Moore et al. 2015), may respond differently. Moreover, it is not possible to determine the dose of protein necessary to stimulate a maximal MPS response from our data. We examined the MPS response to two amounts of protein only. Further study is required to identify a maximal stimulatory protein dose for MPS following whole‐body resistance exercise and to explore if this maximal dose is influenced by LBM in different populations.

## Conflict of Interests

CEL was an employee of GSK Consumer Healthcare during the course of this study. None of the authors had a conflict of interest.
